# Developmental abnormalities in supporting cell phalangeal processes and cytoskeleton in the *Gjb2* knockdown mouse model

**DOI:** 10.1242/dmm.033019

**Published:** 2018-02-01

**Authors:** Sen Chen, Le Xie, Kai Xu, Hai-Yan Cao, Xia Wu, Xiao-Xiang Xu, Yu Sun, Wei-Jia Kong

**Affiliations:** 1 Department of Otorhinolaryngology, Union Hospital, Tongji Medical College, Huazhong University of Science and Technology, Wuhan 430022, China; 2 Department of Ultrasound, Union Hospital, Tongji Medical College, Huazhong University of Science and Technology, Wuhan 430022, China; 3 Institute of Otorhinolaryngology, Tongji Medical College, Huazhong University of Science and Technology, Wuhan 430022, China

**Keywords:** Connexin26, Cochlear development, *Gjb2*, Hearing loss

## Abstract

Mutations in the *GJB2* gene [which encodes connexin 26 (Cx26)] are the most common causes of hereditary hearing loss in humans, and previous studies showed postnatal development arrest of the organ of Corti in different Cx26-null mouse models. To explore the pathological changes and the mechanism behind the cochlear abnormalities in these mice further, we established transgenic mouse models by conditional knockdown of cochlear Cx26 at postnatal day (P) 0 and P8. Auditory brainstem responses were recorded and the morphological features in the organ of Corti were analyzed 18 days after Cx26 knockdown. Mice in the P0 knockdown group displayed severe hearing loss at all frequencies, whereas mice in the P8 knockdown group showed nearly normal hearing. In the P8 knockdown group, the organ of Corti displayed normal architecture, and no ultrastructural changes were observed. In the P0 knockdown group, the phalangeal processes of Deiter's cells did not develop into finger-like structures, and the formation of microtubules in the pillar cells was significantly reduced; moreover, the amount of acetylated α-tubulin was reduced in pillar cells. Our results indicate that *Gjb2* participates in postnatal development of the cytoskeleton in pillar cells during structural maturation of the organ of Corti. In P0 knockdown mice, the reduction in microtubules in pillar cells might be responsible for the failure of the tunnel of Corti to open, and the malformed phalangeal processes might negatively affect the supporting framework of the organ of Corti, which would be a new mechanism of *Gjb2*-related hearing loss.

## Abstract (precis)

**Summary:** A reduction in connexin 26 before opening of the tunnel of Corti impedes microtubule formation in supporting cells, and this may lead to cochlear developmental abnormalities and deafness in the *Gjb2* knockdown mouse model.

## INTRODUCTION

Gap junctions are intercellular channels that connect adjacent cells and provide communication conduits between cells (Bennett et al., 1991). As an intercellular channel, the gap junction has a relatively large pore that allows ions, microRNA, secondary messengers and metabolites ≤1.5 kDa to pass through (Harris, 2001; Zhu et al., 2015b). Gap junctions consist of protein subunits called connexins, which are encoded by a family of homologous genes. Six connexins form a hemichannel, and two hemichannels from a pair of adjacent cells constitute a gap junction. Through their vital roles in cellular communication, connexins have been shown to be required for the differentiation and maturation of different organs, such as the heart, the eye, the skeleton and nervous tissue (Moorer and Stains, 2017; Su et al., 2017; Hu et al., 2017). In the mammalian inner ear, connexin 26 (Cx26, encoded by the *GJB2* gene) is the main connexin, and it assembles into gap junctions in all supporting cells and fibrocytes (Ahmad et al., 2003; Kikuchi et al., 1995; Zhao and Yu, 2006). *GJB2* mutations are the most common cause for hereditary hearing loss in humans and are responsible for about a quarter of all cases of hereditary hearing loss. To date, >100 *GJB2* mutations have been linked to hearing impairment (Chan and Chang, 2014; Rabionet et al., 2000). However, the mechanism through which these mutations lead to deafness is still not well understood.

In studies of *GJB2*-related hearing loss, the common pathologies are the failure of the tunnel of Corti (TC) to open and the disappearance of Nuel's space (NS), and these malformations of the organ of Corti (OC) have been observed both in humans and in transgenic mice (Griffith et al., 2006; Wang et al., 2009). In 2006, a male infant diagnosed with keratitis–ichthyosis–deafness syndrome was reported, which was caused by the *GJB2* G45E heterozygous mutation. In that case, examination of the temporal bone revealed dysplasia of the OC, with undifferentiated cells (Griffith et al., 2006).

The situation in mouse models shares some similarities with humans. In 2008, a Japanese group established a *Gjb2* R75W mutant mouse model showing a closed TC and reduced microtubules in the inner pillar cells (IPCs) (Inoshita et al., 2008). *Gjb2* mutations have diverse effects, and cochlear functions affected by null mutations might directly reflect the functions of Cx26. By using the loxP-Cre system, Lin's group has established three conditional Cx26-null mouse lines in which cochlear Cx26 can be knocked out at different embryonic stages. All these mouse lines suffer from severe hearing loss and closed TC in the OC (Wang et al., 2009). However, this cochlear abnormality has not been considered to be the cause of hearing loss in both human cases and animal models. Generally, small molecule or ion communication disorders, such as potassium recycling defects, have been considered to be the main cause for the congenital hearing loss in *Gjb2* mutation or Cx26-null models (Kikuchi et al., 2000a,b; Zhang et al., 2005).

The mouse inner ear is still immature at birth, and hearing does not reach the adult level until postnatal day (P)16-P18. By using timed conditional knockdown techniques, we and others have reported that hearing and the structure of the OC in 1-month-old mice are not significantly affected when cochlear Cx26 is knocked down at P10 or P12 (Chen et al., 2014; Zhu et al., 2015a); however, mild to moderate hearing loss with a well-developed OC could be observed ∼2 months later. Additionally, we and Lin's group have observed that severe congenital hearing loss and malformed OCs occur in Cx26 knockdown mice only if the cochlear Cx26 knockdown is earlier than P4 (Chang et al., 2015). The most obvious differences between the early (before P4) and late (after P6) Cx26 knockdown mouse models are the developmental arrest in the OC and the extent of deafness (Chang et al., 2015; Chen et al., 2014; Zhou et al., 2016). Considering the above findings, it is difficult to attribute two different hearing loss patterns to the same small molecule or ion communication disorder hypothesis (Zhang et al., 2005; Zhu et al., 2015b). Human *GJB2* mutation cases, *Gjb2* mutation mouse models, embryonic Cx26-null mouse models and early postnatal Cx26 knockdown mouse models all display similar severe hearing loss and malformed OCs. Thus, developmental abnormality might be a better candidate for explaining the severe congenital hearing loss caused by Cx26 downregulation before P4. This early Cx26 knockdown mouse model is likely to imitate the severe congenital hearing loss caused by *GJB2* mutations in humans.

In order to explore the mechanism involved in the developmental arrest of the OC, we induced cochlear Cx26 knockdown in P0 and P8 mice. The TC begins to open at P5, and this process is nearly complete at P8-P9. The opening of the TC is a milestone in the proper development of the structures of the OC. Therefore, the P0 knockdown group (P0 KD group) was used to investigate the role of Cx26 in the immature OC structure, whereas the role of Cx26 function in an open TC was studied in the P8 knockdown group (P8 KD group). Thus, we investigated the cochlear pathology after knocking down Cx26 at two key developmental stages. Abnormal phalangeal processes in Deiter's cells (DCs) and a decrease in the number of microtubules in pillar cells (PCs) were observed in the P0 KD group, indicating that Cx26 participates in the postnatal maturation of phalangeal processes in DCs and the cytoskeleton in PCs. The reduced microtubules in PCs in the presence of reduced Cx26 very early in development might be the reason for the failure of the TC to open, and this and the malformed phalangeal processes seen in DCs might be a potential cause for congenital *GJB2*-related hearing loss.

## RESULTS

### Cx26 knockdown in the cochlea and Cre-positive counts in timed conditional Cx26 knockdown mice

**Fig. 1.**
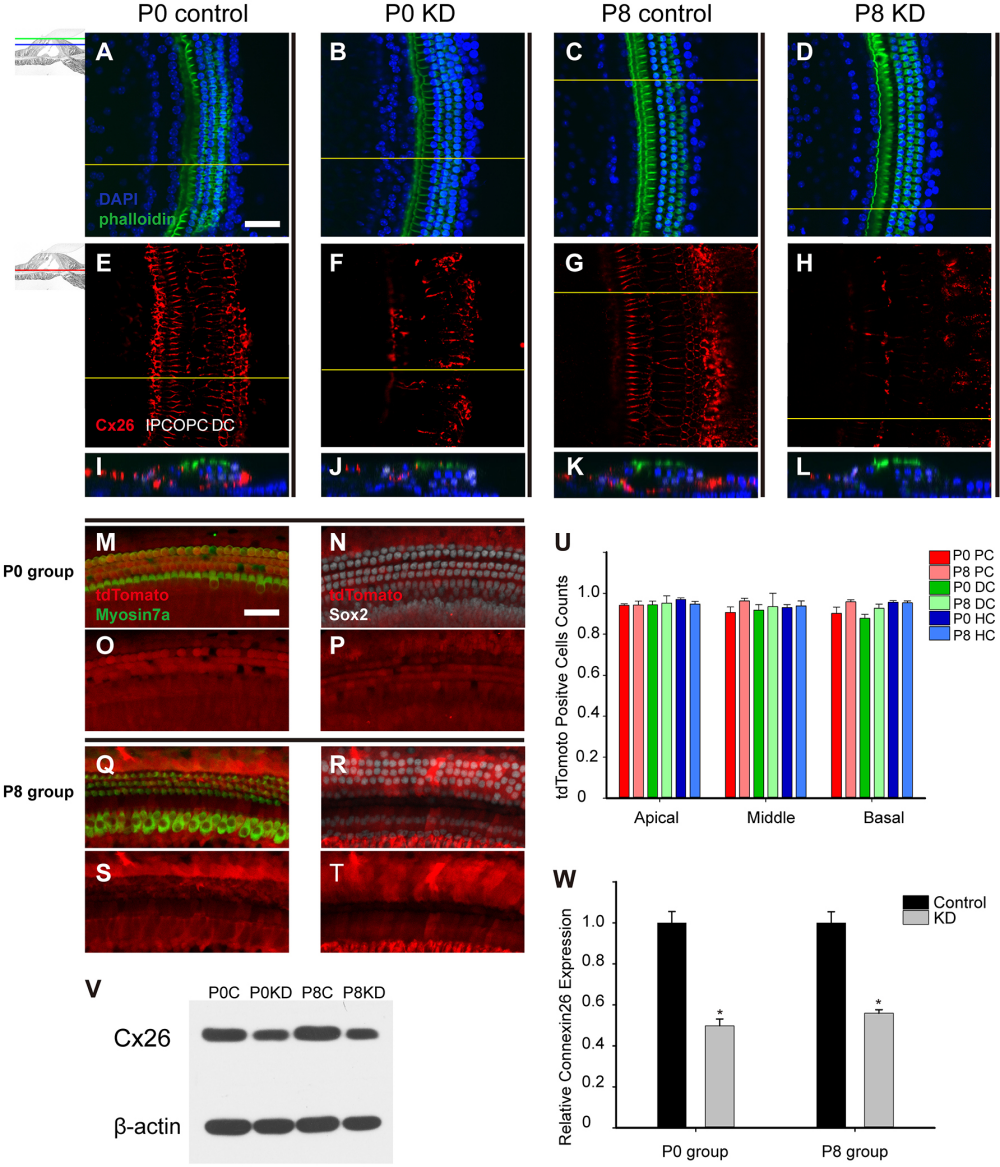
**Cx26 KD in the cochlea and Cre-positive cell counts in timed conditional Cx26 KD mouse models.** Summary of Cx26 expression in control and experimental groups. (A-D) The mice were sacrificed 18 days after TMX injection, and the pictures were captured at the apical turn (20–35% from the apex) from the P0 control group (A), the P0 KD group (B), the P8 control group (C) and the P8 KD group (D). (E-H) Immunolabeling of Cx26 (red) in the stretched preparation in the corresponding control and experimental groups. (I-L) Cross-sections were generated using confocal reconstruction from the corresponding control and experimental groups. The tdTomato signal was detected 5 days after TMX injection. (M,Q) The tdTomato staining (red), with hair cells labeled by Myosin7a (green) in the P0 (M) and P8 (Q) groups. (N,R) The tdTomato staining (red), with supporting cells labeled by Sox2 (white) in the P0 (N) and P8 (R) groups. (O,P,S,T) Single tdTomato signals are shown in the corresponding pictures. (U) The percentages of tdTomato-positive cells in the P0 and P8 groups (*n*=3 in each group). (V) Representative western blot results are shown for the P0 control, P0 KD, P8 control and P8 KD groups; β-actin was used as the control. (W) Relative Cx26 expression (*n*=4 in each group) in the control and experimental groups. The differences between the P0 KD group and the P8 KD group were not significant (*P*>0.05). *Significantly different from the control group (*P*<0.05). Scale bar: ∼50 µm (A,M). DC, Deiter's cells; HC, hair cells; PC, pillar cells.

In this study, Cx26 was successfully knocked down in the P0 KD and P8 KD groups. The images of hair cell staining (DAPI) and stereocilia staining (phalloidin) were merged (Fig. 1A-D). Generally, Cx26 (red) was located along the edges of PCs, DCs and neighboring supporting cells in the control groups (Fig. 1E,G). By contrast, a significant reduction in Cx26 staining was observed in PCs, DCs and other supporting cells from both the P0 KD and P8 KD groups (Fig. 1F,H). In the reconstructed cross-sections, the deletion patterns of the P0 KD and P8 KD groups were nearly the same in the cochlear epithelium (Fig. 1I-L).

To observe the knockdown patterns better, the Cre activation was visualized by tdTomato expression (red). The same tamoxifen (TMX) administration protocol (at P0 or P8) was used in a reporter mouse line to evaluate the Cre activation, and the two groups were defined as the P0 and P8 groups. Images were obtained 5 days after TMX administration. The hair cells labeled by Myosin7a (green) and the supporting cells labeled by Sox2 (white) both had high Cre activation (Fig. 1M,N,Q,R). As shown in Fig. 1U (*n*=3 in each group), the grouped tdTomato-positive cell counts showed similar Cre activation (>89%) in PCs, DCs and hair cells in both the P0 and P8 groups. There were no statistically significant differences between the P0 and P8 groups for Cre activation (*P*=0.173–0.852 in different cell type comparisons between the P0 and P8 groups). Consistent with tdTomato counts, western blot quantification showed nearly the same cochlear Cx26 reduction in the P0 KD and P8 KD groups (Fig. 1V,W). Compared with controls, the Cx26 expression was 49.7±6.6 and 55.9±3.4% in the P0 KD and P8 KD groups, respectively (*n*=4, *P*=0.15).

### Significant hearing loss was observed only in the P0 KD group

**Fig. 2.**
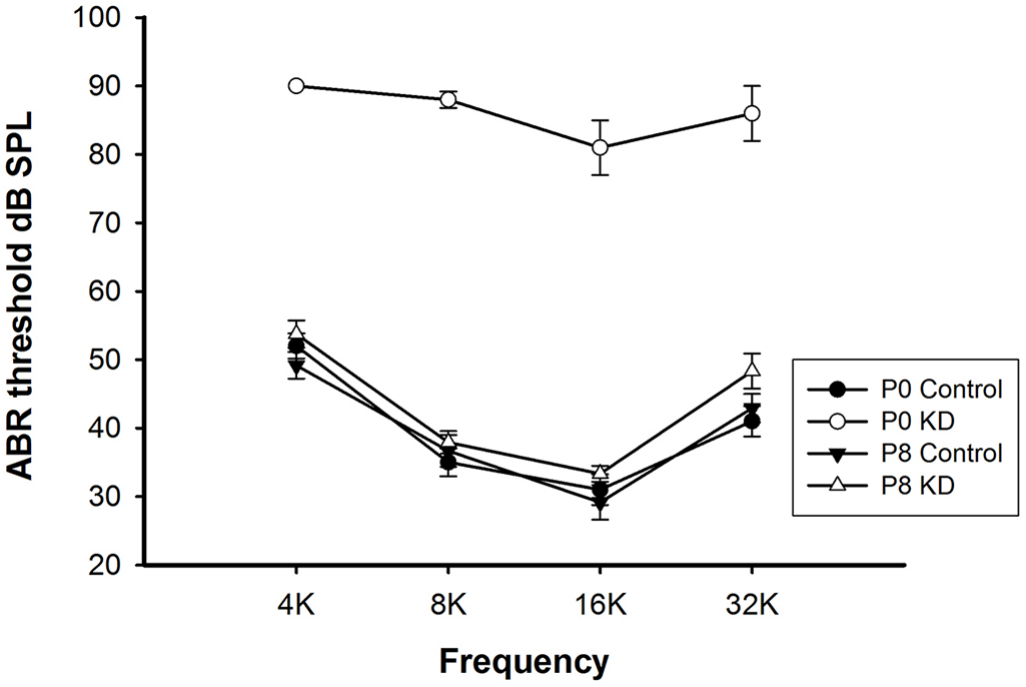
**Significant hearing loss was observed only in the P0 KD group.** Auditory thresholds (*n*=5–6 in each group) were measured in the P0 control, P0 KD, P8 control and P8 KD groups. All ABR tests were performed 18 days after TMX injection.

Hearing in mice matures at P16-P18, and it remains stable for many months according to the different strains. To test the requirement of Cx26 for hearing, auditory brainstem response (ABR) measurements were performed 18 days after TMX injection. Consistent with previous publications, mice in the P0 KD group (*n*=5) showed severe congenital hearing loss (Fig. 2). Thresholds across a range of frequencies were reduced, with certain frequencies having ABR thresholds up to 90 dB SPL. However, the thresholds of the P8 KD group (*n*=6) at 4, 8, 16 and 32 kHz were 53.8±2.0, 37.9±1.7, 33.3±1.2 and 48.3±2.6 dB SPL, respectively. Compared with controls, the ABR thresholds in the P8 KD group showed no significant differences (*P*=0.162–0.675 for the different frequencies).

### Developmental arrest of the OC occurred only when Cx26 was knocked down at the early postnatal stage

**Fig. 3.**
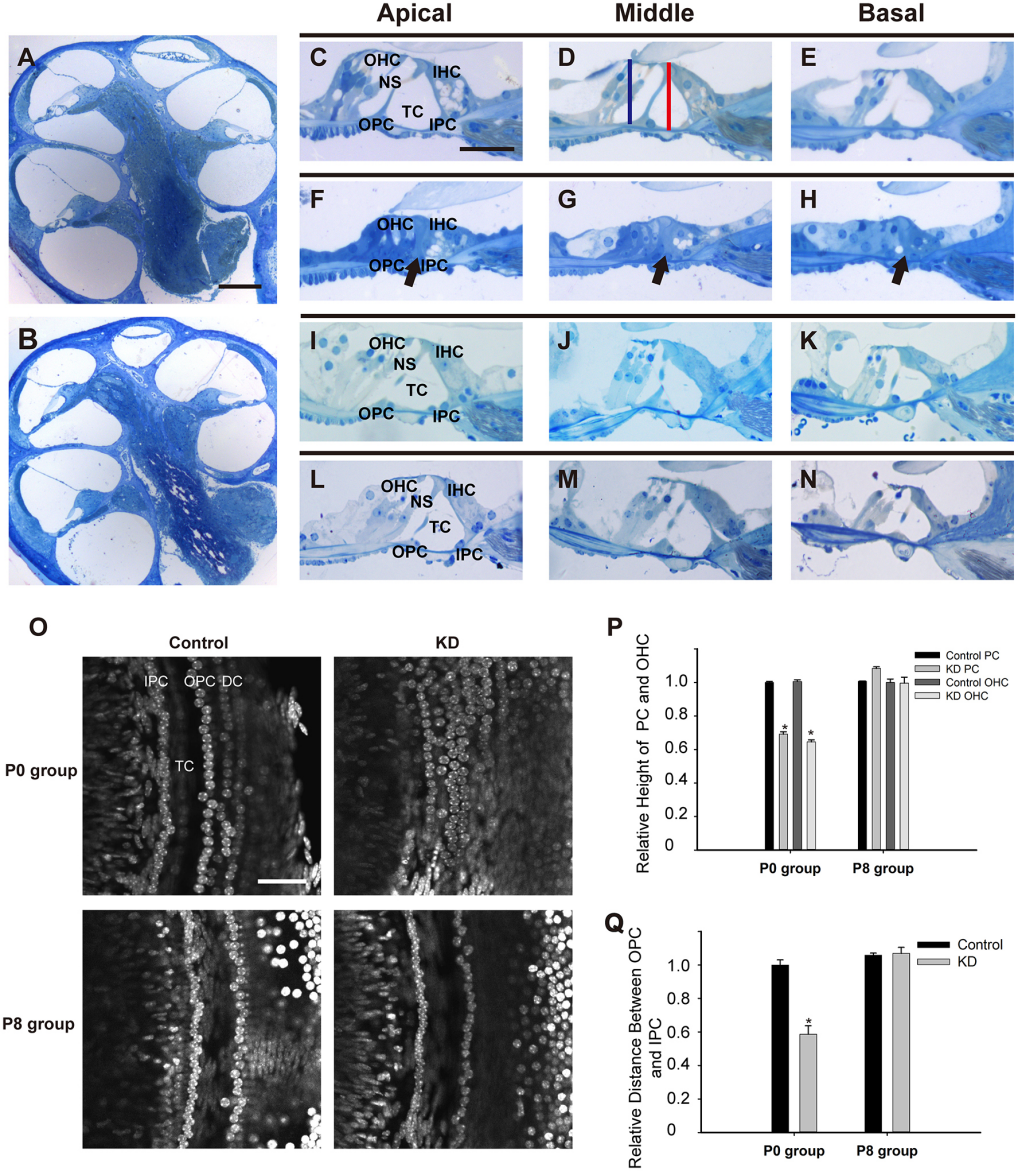
**Developmental arrest of the OC occurred only when Cx26 was knocked down at the early postnatal stage.** (A,B) A full view of the cochlear sections obtained from the P0 control (A) and P0 KD groups (B). (C-E) Morphology of the OC in the apical, middle and basal turns from the P0 control group. The red line in panel D indicates the distance from the head conjunction of the IPCs and OPCs to the basilar membrane, and the blue line shows the distance from the top of the third row of OHCs to the basilar membrane. (F-H) Morphology of the OC in different turns from the P0 KD group. The black arrows indicate the closed TCs in different turns. (I-K) Morphology of the OC in different turns from the P8 control group. (L-N) Morphology of the OC in different turns from the P8 KD group. (O) The stretched preparation at the supporting cell level shows the distance between the IPCs and OPCs in the control and experimental groups. (P) Relative heights of the PCs and the third row of OHCs in the control and experimental groups. (Q) Relative distances between the IPCs and OPCs in the control and experimental groups. *Significantly different from the control group (*P*<0.05). Scale bar: ∼200 µm (A); ∼40 µm (C,O).

Postnatal development of the OC is a dynamic process that involves the increasing height of the OC and the opening of the TC. The TC usually opens completely in all turns at P8-P9, and the cytoskeletal structure matures at P18 (Roth and Bruns, 1992a,b). The milestone event in OC development is the formation of the triangular structure by IPCs and outer pillar cells (OPCs), which helps to stabilize the OC (Zetes et al., 2012). Therefore, morphological observations were performed 18 days after TMX administration in both P0 and P8 mice. In the P0 KD group, the height of the OC was significantly reduced in the apical, middle and basal turns. Additionally, the hair cells and adjacent PCs and DCs remained in a compressed state in all three turns (Fig. 3F-H). The failure of the TC to open and lack of NS in the P0 KD group hindered the formation of the normal spatial structure (black arrow, Fig. 3F-H). By contrast, the OC in the P8 KD group showed normal organization with a well-formed NS that was similar to controls in all three turns (Fig. 3L-N). Compared with controls, the distance between the head conjunction of the IPCs and OPCs and the basilar membrane was reduced to 69.2±1.4% (*n*=3, *P*<0.001), and the distance between the head of the third row of outer hair cells (OHCs) and the basilar membrane was reduced to 64.5±1.2% (*n*=3, *P*<0.001) in the P0 KD group (Fig. 3P). Another remarkable change was the gathering of the IPC and OPC nuclei in the P0 KD group, and the distance between them was reduced to 58.7±5% (*n*=3, *P*<0.001) when compared with controls (Fig. 3O,Q). However, no significant differences were seen in the P8 KD group (*n*=3, *P*=0.791).

### Malformed phalangeal processes of DCs in the P0 KD group

**Fig. 4.**
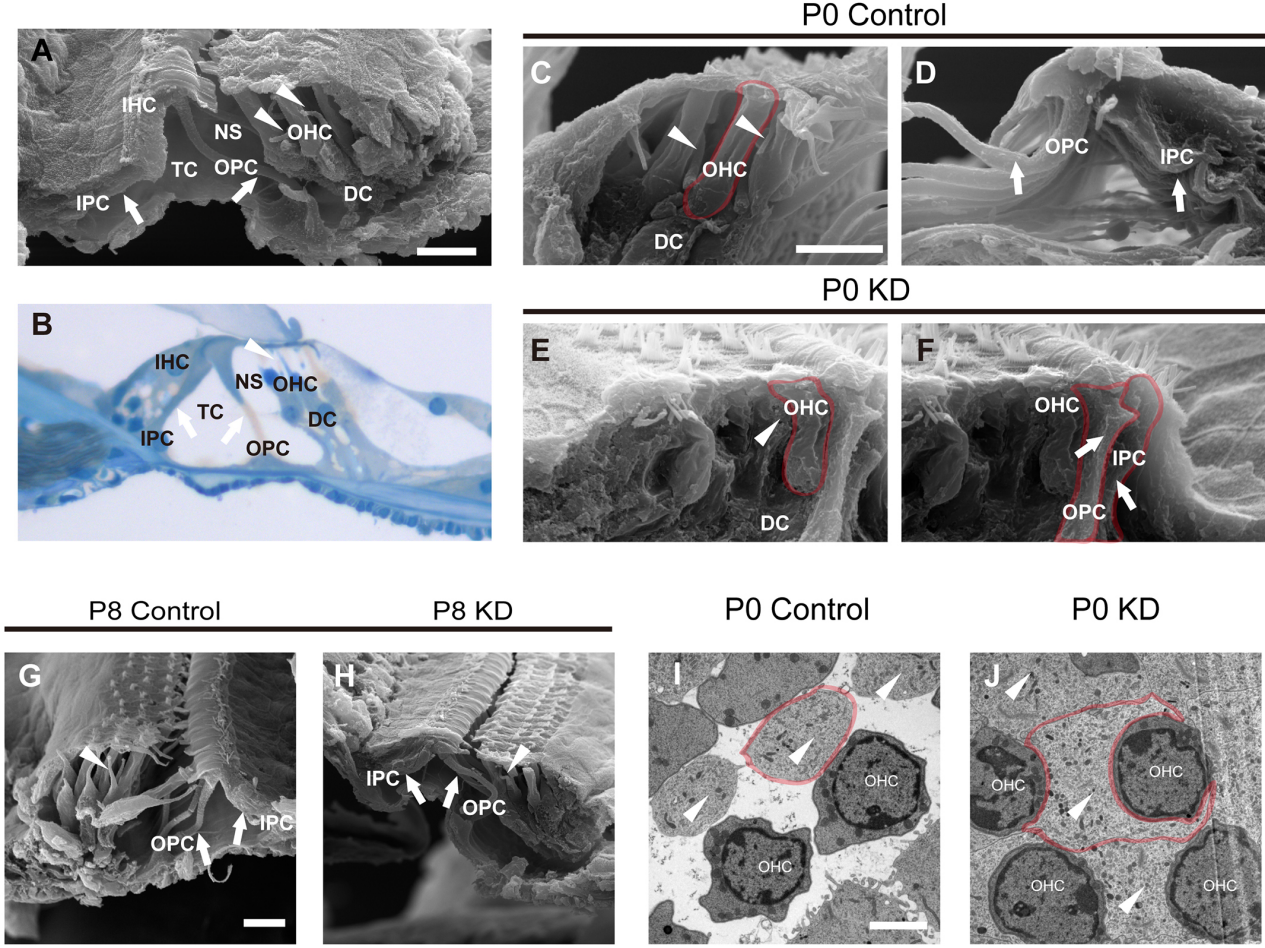
**Spatial structures of the OC visualized by SEM in the P0 control, P0 KD, P8 control and P8 KD groups.** (A) A full view of the OC was scanned by SEM from a control mouse at P30. The white arrows indicate PC bodies. The white arrowheads indicate the normal morphology of the phalangeal processes from the DCs. (B) A full view of an OC section from a control mouse at P30 stained with Toluidine Blue. The white arrows and the arrowhead indicate PC bodies and the phalangeal process of a DC, respectively. (C) Magnified image showing the thin and long phalangeal processes of DCs (white arrowheads) located between OHCs from the P0 control group, and NS was well formed between the OHCs and the phalangeal processes. The boundary of an OHC is shown by the red line. (D) The white arrows indicate mature OPCs and IPCs from the P0 control group, and the triangular TC is well formed. (E) The malformed phalangeal process of a DC (white arrowhead) in contact with an OHC. NS was not present in the P0 KD group. The boundary of an OHC is shown by the red line. (F) The OPC and IPC (white arrows) grew snugly together, and the TC never formed in the P0 KD group. The boundaries of an OPC and an IPC are shown by the red line. (G,H) The OCs from P8 control (G) and KD groups (H) were visualized by SEM, and the PCs (white arrows) and DCs (white arrowheads) were well developed in both groups. (I) A horizontal section from the P0 control group was captured at the OHC level by TEM, and the phalangeal processes of the DCs (white arrowheads) were not in contact with the OHCs. The boundary of one of the DC's phalangeal processes is shown by the red line. (J) The hypertrophic phalangeal processes of the DCs (white arrowheads) embraced the OHC bodies in the P0 KD group, and the boundary of one of the DC's phalangeal processes is shown by the red line. Scale bar: ∼30 µm (A,C,G); ∼5 µm (I).

The phalangeal processes of DCs are important for maintaining the structure of the OC. Fig. 4A,B shows the mature OC at P30 visualized by scanning electron microscopy (SEM) and light microscopy, respectively. Parts of the body of the PC have developed into thin, long, column-like structures (white arrows in Fig. 4A,B) that are separate from each other to form the TC. The phalangeal processes of the DCs are finger-like structures (white arrowheads in Fig. 4A,B). The SEM images provide more details of the spatial structure than traditional stained sections (Fig. 4A). According to a previous study and our observation, the mice in the P0 KD group might have a small amount of hair cell loss in the middle turn at P18, and this could hinder the observation of very small structures (Wang et al., 2009). Thus, the apical turn of the OC was prepared for further observation. All observations were performed 18 days after TMX administration in both the P0 and P8 groups. The OC in the P0 control group shared the same spatial structure organization as the controls at P30 (Fig. 4C,D). Unlike the PCs, the phalangeal processes of the DCs (white arrowheads in Fig. 4C, with more details in Fig. S1) reached diagonally from near the bottom of one hair cell to the reticular lamina near the top of another hair cell. Unexpectedly, the abnormal phalangeal processes of the DCs (white arrowhead in Fig. 4E) lost their shape in the P0 KD group and adhered to the OHC body, while IPCs and OPCs came into contact with each other (labeled by a red line in Fig. 4F). By contrast, the phalangeal processes of the DCs in the P8 KD group were well shaped and organized in a similar manner to those in the P8 control group (white arrowheads in Fig. 4G,H), and the triangular TC was well formed by a pair of IPCs and OPCs (white arrows in Fig. 4G,H). To investigate the disappearance of NS, horizontal sections through the hair cell level were visualized by transmission electron microscopy (TEM). It was notable that hypertrophic phalangeal processes of DCs in the P0 KD group embraced the OHC body and occupied the remaining space between the OHCs (white arrowheads in Fig. 4J,I). The reduced cytoplasm of the OHCs indicated that the hypertrophic phalangeal processes might squeeze the OHCs in the P0 KD group.

### Microtubule formation decreased in PCs from the P0 KD group

**Fig. 5.**
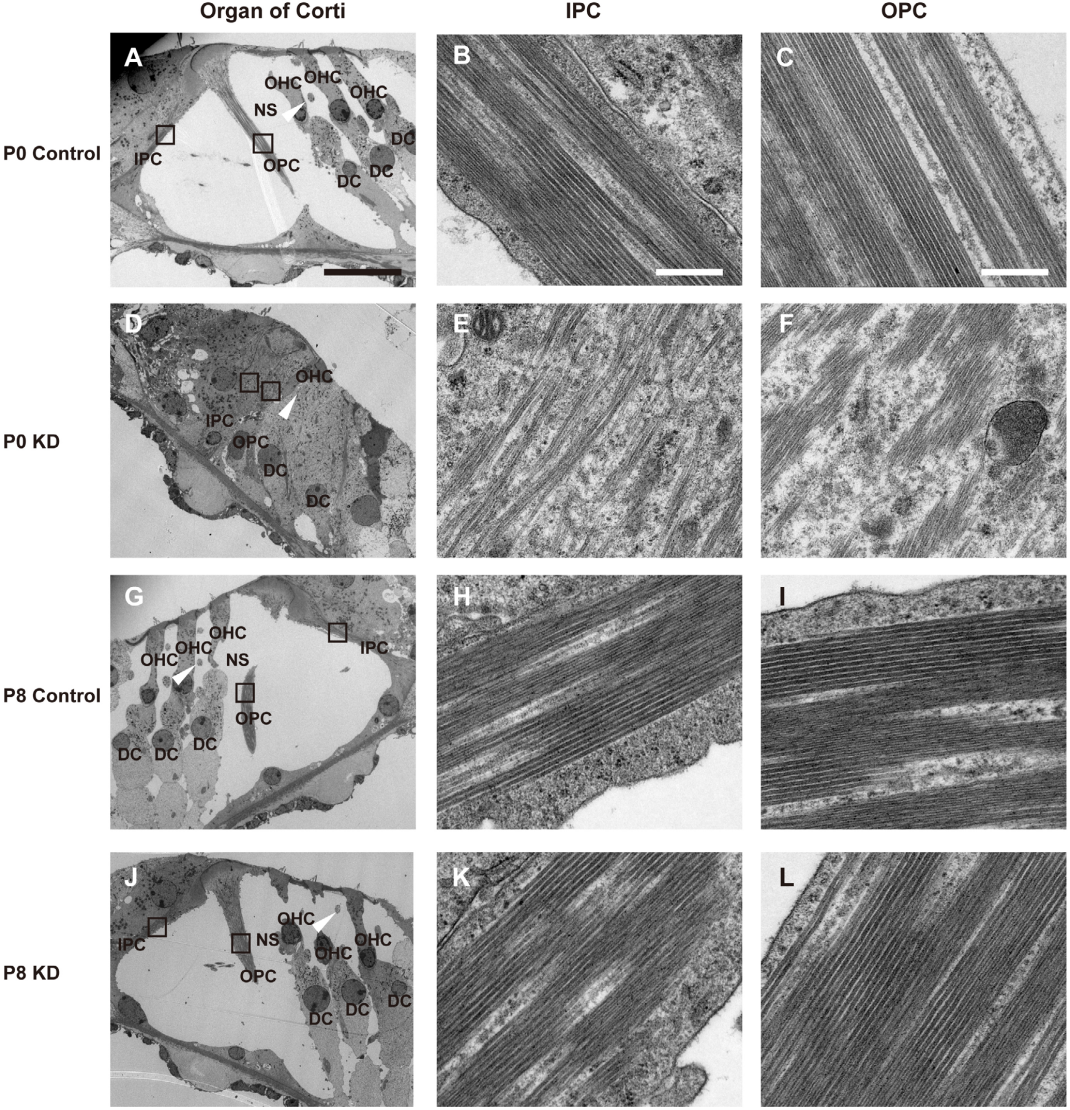
**Ultrastructure of the apical turn of the OC from the P0 control, P0 KD, P8 control and P8 KD groups.** (A) A full view of the OC visualized by TEM in the P0 control group. The white arrowhead indicates a phalangeal process of the first-row DC. (B,C) The black boxes in panel A are magnified to show the pattern and arrangement of microtubules in the IPC (B) and OPC (C) from the P0 control group. The bundles of microtubules run parallel with the long axis of the PCs, and there are nearly no organelles visible in the cytoplasm. (D) A closed TC from the P0 KD group. The white arrowhead indicates a hypertrophic phalangeal process of the first-row DC. (E,F) The magnified images show the details of the microtubules in an IPC (E) and OPC (F). The microtubules in the IPC and OPC were scattered, and some of the microtubules had an arched shape. (G,J) The mature OCs are shown from P8 control (G) and P8 KD (J) groups, respectively. The white arrowheads indicate the corresponding phalangeal processes of the DCs. (H,I) Magnified images of the microtubules in an IPC (H) and OPC (I) from the P8 control group. (K,L) Magnified images of the microtubules in an IPC (K) and OPC (L) from the P8 KD group. Scale bar: ∼20 µm (A); ∼500 nm (B,C).

Microtubules are extensively expressed in supporting cells, especially in PCs and DCs, and they play an essential role in maintaining the stiffness of the sensory epithelium. As shown in Fig. 5A, bundles of microtubules were mainly formed in the PCs, and the white arrowhead indicates a differentiated phalangeal process from a DC. The black boxes in Fig. 5A are magnified in Fig. 5B,C to show the parallel arrangement of microtubules in the IPCs and OPCs, respectively, from the P0 control group. The enlarged DCs with abnormal phalangeal processes (white arrowhead in Fig. 5D) sustained the reticular lamina in the P0 KD group. Compared with the control group, the numbers of microtubules in PCs from P0 KD mice were significantly reduced. Moreover, the microtubules were relatively scattered, and some formed an arched shape and were no longer linear. The microtubules decreased significantly in the IPCs of the P0 KD group, whereas there were still a few bundles of microtubules in the OPCs. In the mature PC body, it was difficult to observe any organelles other than microtubules in the cytoplasm, but when the TC was still closed (before P4) many organelles, such as mitochondria, Golgi bodies and ribosomes, could be observed in the PC bodies (data not shown). However, different cell organelles, such as mitochondria and Golgi bodies, were still observed in the cytoplasm in the PC body from the P0 KD group. This indicated that the PCs in the P0 KD group might still be at a juvenile stage. The framework of the OC developed normally in the P8 KD group (Fig. 5G,J), and in the P8 KD group the pattern and the density of the microtubules showed a similar arrangement to that of controls (Fig. 5K,L). Similar to the controls, it was difficult to see any organelles in the PC body from the P8 KD group.

### Cytoskeleton developmental arrest was seen in PCs from the P0 KD group

**Fig. 6.**
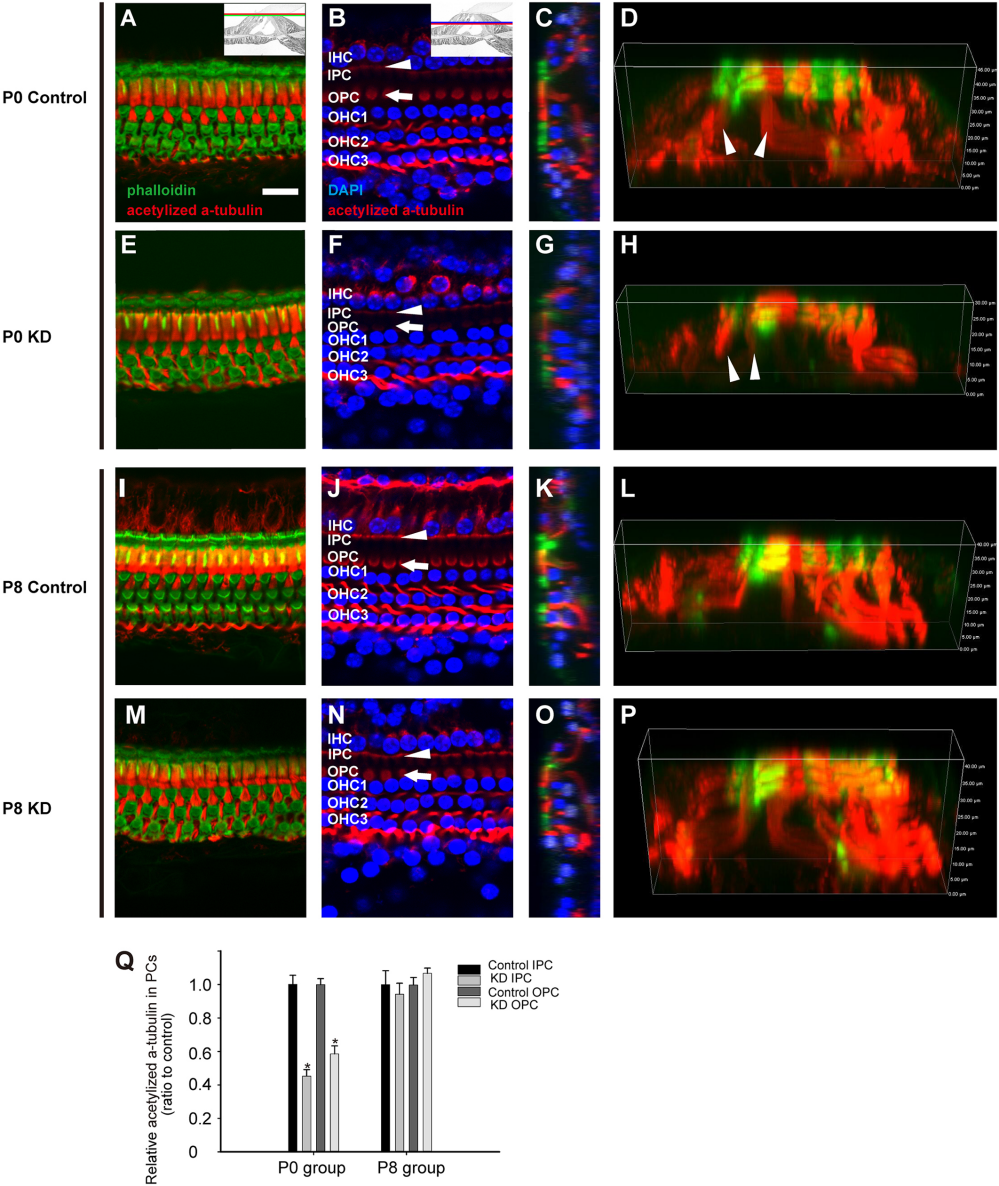
**Cytoskeleton staining in the OC from the P0 control, P0 KD, P8 control and P8 KD groups.** (A,E,I,M) F-actin (green) and acetylated α-tubulin (red) staining at the cuticular plate level in the P0 control, P0 KD, P8 control and P8 KD group, respectively. (B,F,J,N) F-actin (green) and acetylated α-tubulin (red) staining and the hair cell nuclei (blue) level in the P0 control, P0 KD, P8 control and P8 KD group, respectively. The positions of different cells are indicated. The white arrowheads point out the bodies of IPCs, and the white arrows point out the bodies of OPCs. (C,G,K,O) Cross-sections were generated to show the cytoskeletal patterns in the different groups. A triangular TC can be observed in the different groups, except for the P0 KD group in panel G. (D,H,L,P) The spatial arrangement of the cytoskeleton of the OC was reconstructed to show details in the different groups. (Q) Quantification of acetylated α-tubulin fluorescence in PCs at the level of the hair cell nuclei (*n*=3 in each group). *Significantly different from the control group (*P*<0.05). Scale bar: ∼30 µm (A).

Previous studies showed that acetylated α-tubulin is a specific marker for the microtubules in supporting cells (Bane et al., 2002; Slepecky et al., 1995). Consistent with previous papers, immunofluorescent staining of acetylated α-tubulin was observed in PCs and DCs (Fig. 6D,L). The PCs were intensely labeled, especially at the apical surface and at the hair cell level (Fig. 6A,I). In the P0 KD mice, the acetylated α-tubulin labeling in the IPC and OPC bodies at the hair cell level was significantly reduced (white arrowhead and white arrow in Fig. 6F). Although the acetylated α-tubulin was dramatically reduced in the P0 KD mice, the F-actin (green) in the reticular lamina remained stable (Fig. 6E). Consistent with the SEM findings, the three-dimensional reconstruction image in the P0 KD group showed a low OC with less acetylated α-tubulin staining in PCs than that of controls (white arrowhead in Fig. 6D,H). Not surprisingly, the acetylated α-tubulin staining in the P8 KD group displayed a similar pattern to that in the P8 control group (Fig. 6L,P). Compared with controls, quantification of acetylated α-tubulin fluorescence at the hair cell level showed that the relative fluorescence in IPCs and OPCs from the P0 KD group decreased to 45.28±3.85 and 58.58±4.76%, respectively (*n*=3, *P*<0.01). However, the relative fluorescence in IPCs and OPCs from the P8 KD group showed no statistical differences when compared with controls (*n*=3).

## DISCUSSION

Cx26 is suggested to play significant roles in OC structural development, and the deformities of OCs in all turns might be a potential cause for the severe hearing loss in the P0 KD group. In this study, a well-developed OC is the fundamental prerequisite for the good hearing in the P8 KD mice. Although cochlear Cx26 was successfully downregulated in the P8 KD mice, the ultrastructure of PCs and DCs was barely affected. This observation indicated that downregulation of cochlear Cx26 does not hinder normal hearing in a fully developed OC. By contrast, mice in the P0 KD group suffered hearing loss across all frequencies. Generally, the dysfunction or death of hair cells has been considered the most common cause of hearing loss. A previous study in conditional Cx26-null mice indicated that the maturation of ribbon synapses in IHCs and the postnatal refinement process of type I fibers are affected (Chang et al., 2015). However, another study indicated that electromotility of isolated OHCs was preserved in Cx26 R75W mutant mice (Minekawa et al., 2009). According to previous studies, the hair cells and spiral ganglion cells do not express Cx26 protein (Zhao and Santos-Sacchi, 1999; Zhao and Yu, 2006). Therefore, the changes in hair cells or type I fibers mentioned above might be a secondary effect caused by downregulation of Cx26 in supporting cells. Further investigations are needed to determine whether the hearing loss was caused directly by deformities of OCs in the P0 KD group.

The developmental abnormality in PCs might be the fundamental factor for failure of the TC to open. In the control group, the TC in the different turns of the OC began to open at P5-P9. During this process, the juvenile PCs changed from a compressed state into a separated and thin state. The elongation, thinning and separation of IPC and OPC cell bodies changed the TC from a closed to an open state. In the P0 KD group, the PC structural differentiation was prevented during early postnatal development, and lower height and nuclear mispositioning were observed in PCs. These findings indicate that cochlear Cx26 is involved in the postnatal differentiation and maturation of PCs. In the P8 KD group, the spatial structure and the relative position of the IPCs and OPCs matured normally during postnatal development. These observations indicated that a reduction in cochlear Cx26 after P8 does not impede the differentiation of PCs.

In our study, downregulation of Cx26 before TC opening might impede the formation and arrangement of microtubules in PCs, which would be a likely cause for the arrest of PC differentiation. Bundles of microtubules are the typical feature of PCs, and it is believed that these are the mechanical foundation for the supporting function of PCs (Tucker et al., 1995; Zetes et al., 2012). Moreover, microtubules composed of different tubulins have been shown to be the main component of the cytoskeleton in mature PCs (Jensen-Smith et al., 2003; Slepecky et al., 1995). During murine PC differentiation, a small number of the microtubules developed in PCs before TC opening (before P4). When the TC began to open at P5-P8, the density of microtubules was increasing in PCs. Additionally, bundles of microtubules could be observed in PCs at P9 when the TC was fully open (Fig. S2). These observations indicated that the microtubule provides the structural support for the TC opening. In the P0 KD group, a significant reduction of microtubules was observed in both IPCs and OPCs. Some microtubules had an arched shape and lost their polarity. However, the microtubules and their arrangement were barely affected in the P8 KD group. These observations indicated that cochlear Cx26 is involved in microtubule formation and arrangement during PC differentiation and maturation. Once the bundles of microtubules were well developed in PCs, Cx26 was not needed for the maintenance of the microtubules. Another study in *Gjb2* R75W mutant mice found that the microtubules in PCs were significantly reduced at P12 (Inoshita et al., 2008). These results indicate that microtubule reduction in PCs might be a common pathological process in both Cx26 knockdown and Cx26 mutation mouse models. Owing to their crucial role in maintaining cell size and shape, the loss of cytoskeletal elements such as microtubules might explain the low height of the OC in the P0 KD group, and adequate microtubule formation in PCs might ensure the normal height of the OC in the P8 KD group.

Microtubules consist of polymerized filaments of α- and β-tubulin monomers. The α-tubulin can be modified post-translationally by acetylation at Lys40, and this modification is believed to be a marker of stable microtubules (Janke and Bulinski, 2011). In the rodent cochlea, acetylated α-tubulin is mainly expressed in PCs and DCs (Slepecky et al., 1995; Szarama et al., 2012). In the P0 KD group, there was a significant reduction of acetylated α-tubulin staining in PCs. This indicated that stable microtubules might not have developed in the PCs, which was consistent with our observations of the ultrastructure. However, the staining of the acetylated α-tubulin in the P8 KD group was nearly the same as in controls. This indicated that a reduction in acetylated α-tubulin might be responsible for the reduction of microtubules in PCs of the P0 KD group.

The most interesting finding in the present study was the hypertrophic phalangeal processes of DCs in the P0 KD group, and we hypothesize that this is another pathological basis for the hearing loss observed in these mice. DC differentiation is a dynamic process, and the typical character of DCs is that parts of the compressed and cylindrical cell bodies develop into finger-like structures during postnatal development. These phalangeal processes separate from each other during postnatal development, and the space created between the phalangeal processes and OHCs is referred to as NS. The phalangeal processes of the DCs provide structural support for the OHCs, and NS is filled with cortilymph. In the present study, the shortened phalangeal processes did not develop into finger-like structures, and they lost their polarity in the P0 KD group. Consequently, NS and the cortilymph were never formed. Moreover, the bodies of the OHCs were squeezed by the hypertrophic phalangeal processes. The cortilymph contributes to the driving force underlying OHC depolarization and some exchange of material (Zdebik et al., 2009). Therefore, the disappearance of cortilymph will lead to the failure of material exchange between the OHCs and the cortilymph, which might be another potential cause for the severe hearing loss seen in the P0 KD group. Not surprisingly, the spatial structure of the DCs was barely affected in the P8 KD group. This finding indicated that early cochlear Cx26 expression is involved in DC differentiation and that the maintenance of the mature DC spatial structures does not depend on cochlear Cx26.

In our study, we systematically investigated the postnatal development of the OC in P0 KD and P8 KD mice. Although cochlear Cx26 expression was largely reduced in P8 KD mice, the postnatal morphology of the OC was very similar to that of controls. However, the microtubules in the PCs of the P0 KD group were severely disrupted, and this might lead to disruptions in the overall framework of the OC. Additionally, the hypertrophic phalangeal processes of the DCs seen in the P0 group provide direct evidence that Cx26 participates in the maturation of phalangeal processes. Taken together, our results indicate that *Gjb2* might be a primary regulator of cytoskeleton formation during postnatal development of the supporting cells of the OC and that Cx26 functions only in early OC development, before P8. In conclusion, the present study demonstrated that loss of cochlear Cx26 in early postnatal stages can lead to malformed structures in the OC, and these specific deformities might be a potential mechanism for the severe deafness in Cx26-null mice.

## MATERIALS AND METHODS

### Mouse models

Cx26^loxP/loxP^ mice and Rosa26CreER mice were provided by Professor Xi Lin at Emory University. TMX (T5648-1G; Sigma-Aldrich, USA)-inducible Cx26^loxP/loxP^;Rosa26CreER mice were generated by crossbreeding of the two strains of mice. Mouse genotyping was performed by PCR amplification of tail genomic DNA. The primer pairs for the Cx26 floxed allele and Cre were as follows: Cx26F 5′-ACAGAAATGTGTTGGTGATGG-3′, Cx26R 5′-CTTTCCAATGCTGGTGGAGTG-3′, CreF 5′-AGCTAAACATGCTTCATCGTCGGTC-3′ and CreR 5′-TATCCAGGTTACGGATATAGTTCATG-3′. Details of the mice were given in our previous paper (Sun et al., 2009; Zhou et al., 2016). To obtain the conditional Cx26 KD mice, we injected TMX (0.75 mg/10 g body weight, s.c., once a day on 2 days consecutively) in the P0 KD and P8 KD groups. We also injected the same amount of TMX in the littermates without the Cre sequence as controls (P0 control and P8 control).

The mouse line tdTomato (ROSA26^CAG-tdTomato−/+^) was provided by Professor Renjie Chai at Southeast University (Madisen et al., 2012). To confirm the Cre expression, we crossed Rosa26CreER mice with tdTomato mice, and the same TMX injection protocol was used in this mouse line (the P0 and P8 group).

All mice were raised in the specific pathogen-free Experimental Animal Center of Huazhong University of Science and Technology. All experimental procedures were conducted in accordance with the policies of the Committee on Animal Research of Tongji Medical College, Huazhong University of Science and Technology (Permit No. 534).

### Auditory brainstem response

ABR was performed 18 days after TMX injection. Mice (*n*=5–7 in each group) were anesthetized with ketamine (120 mg/kg i.p.) and chlorpromazine (20 mg/kg i.p.). A heating pad was used to maintain the body temperature during the test in a soundproof chamber. The positive electrode was inserted at the vertex of the skull, and the reference electrode was placed at the tested ear, with an earth electrode placed at the contralateral ear. A loudspeaker (MF-1; Tucker-Davis Tech., USA) connected to the Tucker-Davis Technologies (TDT) system (RZ6; Tucker-Davis Tech., USA) was placed 10 cm away from the tested ear, with the contralateral ear plugged. Tone burst stimuli were generated, and the responses were recorded by the TDT system. The responses were recorded as the average response to 1024 stimuli and were recorded in decreasing 10 dB steps, which narrowed to 5 dB steps near the threshold. The lowest sound level that could be recognized was considered to be the auditory threshold.

### Protein extraction and western blots

Mice were sacrificed 18 days after TMX injections. Mouse cochleae (*n*=4 in each group) were carefully dissected in ice-cold 0.01 M PBS, and samples of membranous labyrinth were processed in RIPA lysis buffer (P0013B; Beyotime Biotechnology, PR China). The protein concentrations were determined with a BCA Protein Assay Kit (P0012S; Beyotime Biotechnology).

Proteins were separated by electrophoresis on 12% SDS-PAGE gels and transferred to polyvinylidene difluoride membranes. The samples were blocked in TBST (Tris-buffered saline with 0.1% Tween 20) containing 5% milk for 1 h and then incubated with rabbit polyclonal antibodies against Cx26 (1:1000 dilution; 512800; Invitrogen, USA) or rabbit polyclonal antibodies against β-actin (1:1000 dilution; 04-1116; Millipore, USA). Immunodetection was performed with horseradish peroxidase-conjugated goat anti-rabbit antibody and visualized with an ECL reaction kit (P0018; Beyotime Biotechnology). Bands were observed by exposure on X-ray film and analyzed by normalizing to the corresponding β-actin bands using Quantity One 4.6.2 Software (Bio-Rad Laboratories Inc., USA).

### Cochlear tissue preparation and immunofluorescent labeling

Eighteen days after TMX injection, mice were deeply anesthetized by i.p. injection with a combination of ketamine and xylazine, and the cochleae were carefully dissected from the temporal bones and fixed in 4% paraformaldehyde in 0.01 M PBS at room temperature for 1 h. The apical stretched preparation was carefully dissected from freshly dissected cochleae in 0.01 M PBS. The flattened cochlear preparations were incubated in a blocking solution (10% donkey serum with 0.1% Triton X-100) for 1 h at room temperature. The tissue was then incubated with polyclonal rabbit anti-Cx26 antibodies (1:200 dilution; 512800; Invitrogen), polyclonal rabbit anti-myosin7a antibody (1:500 dilution; 25-6790; Proteus BioSciences, USA), monoclonal rabbit anti-α-tubulin antibodies (1:200 dilution; ab179484; Abcam, UK), and polyclonal goat anti-sox2 antibodies (1:100 dilution; sc-17320; Santa Cruz Biotechnology, USA) diluted in 0.01 M PBS with 0.3% Triton X-100 overnight at 4°C. Tissues were washed in 0.01 M PBS with 0.1% Tween-20 and were stained by Alexa Fluor 647 donkey anti-rabbit IgG or Alexa Fluor 594 donkey anti-goat IgG (1:200 dilution; ANT032 and ANT031; antgene, PR China) for 1 h. DAPI (C1005; Beyotime Biotechnology) and phalloidin (0.05 mg/ml; P5282; Sigma, USA) were used for nucleus and F-actin staining, respectively. Images were obtained with a laser scanning confocal microscope (Nikon, Japan). The distance between the nuclei of the IPCs and outer OPCs was measured with Image-Pro Plus 6.0, and the three-dimensional reconstruction images were produced with the NIS-Element software (Nikon, Japan). The immunolabeling of acetylated α-tubulin was quantified from original images, each taken at ×60 magnification in identical conditions. The images were analyzed with ImageJ software, and the relative fluorescence was quantified by normalizing the ratio of average fluorescence of PCs in the KD group (*n*=3) to the average fluorescence of PCs in the corresponding control group (*n*=3).

### Resin sections and transmission electron microscopy

Mice were sacrificed 18 days after TMX injections. The cochleae (*n*=3 in each group) were opened at the apex and fixed in a mixture of 2% paraformaldehyde and 2.5% glutaraldehyde in 0.1 M PBS. The samples were decalcified for 48–72 h in 10% disodium EDTA (pH 7.2) and post-fixed for 1 h in 1% osmium tetroxide. After dehydration through a graded ethanol series, samples were embedded in resin. The samples were sectioned (1.5 μm in thickness) and stained with Toluidine Blue (89640-5G; Sigma-Aldrich, USA) for light microscopy observation and measurement. The distance from the head conjunction of the IPCs and OPCs to the basilar membrane and the distance from the top of the third OHCs to the basilar membrane were measured by Image-Pro Plus 6.0. The ultrathin sections were stained with uranyl acetate and lead citrate for electron microscopy examination (FEI Tecnai G2 20 TWIN; Thermo Fisher Scientific, USA). The middle part of the PCbody was magnified ×9600 and captured by TEM.

### Scanning electron microscopy

Mice were sacrificed 18 days after TMX injections. After fixation and decalcification, the bony capsule and lateral walls were carefully removed from the cochleae to expose the basilar membrane, and the basilar membranes were cut to show the cross-section of the OC. Specimens were dehydrated in a graded series of ethanol, dried (HCP-2, Critical Point Dryer; Hitachi, Japan), mounted on stubs, and sputter-coated with a layer of gold (Eiko Engineering, Japan). Photographs were taken using a scanning electron microscope (VEGA 3 LMU; Tescan, Czechoslovakia).

### Statistical analysis

All data are presented as means±s.e.m. and plotted using Sigma Plot (Version 12.5; Systat Software, Inc., USA). One-way ANOVA with LSD correction or *t*-tests were performed in SPSS software (version 19; IBM SPSS Statistics, USA), and *P*<0.05 was considered to be statistically significant.

## Supplementary Material

Supplementary information
