# Supplementary material for: Developmental abnormalities in supporting cell phalangeal processes and cytoskeleton in the Gjb2 knockdown mouse model
Source: Dis Model Mech. 2018 Feb 1;11(2):dmm033019. doi: 10.1242/dmm.033019 (PMC5894950; doi:10.1242/dmm.033019)
Supplement: Supplementary information [file dmm-11-033019-s1.pdf]

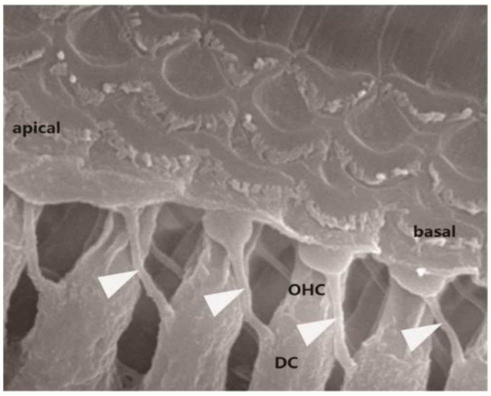

Figure S1. The structure of phalangeal processes in matured DCs. The phalangeal processes of the DCs are finger-like structures labeled by the white arrowhead.

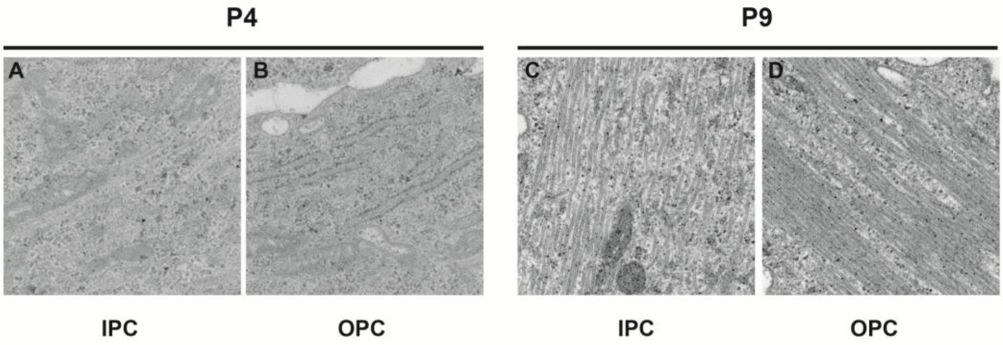

Figure S2. The microtubules of IPCs and OPCs in normal mice at P4 and P9. (A and B): The ultrastructure of an IPC (A) and OPC (B) at P4. (C and B): The ultrastructure of an IPC (A) and OPC (B) at P9. The TEM pictures were captured at 15,000×.

Disease Models & Mechanisms • Supplementary information
